# Current status of antenatal care of pregnant women—8 provinces in China, 2018

**DOI:** 10.1186/s12889-021-11154-4

**Published:** 2021-06-14

**Authors:** Wenling Hu, Huanqing Hu, Wei Zhao, Aiqun Huang, Qi Yang, Jiangli Di

**Affiliations:** National Center for Women and Children’s Health, Chinese Center for Disease Control and Prevention, No.12, Dahuisi Road, Beijing, 100081 China

**Keywords:** Antenatal care, Current status, Pregnant women, China

## Abstract

**Background:**

Antenatal care (ANC) played a crucial role in ensuring maternal and child safety and reducing the risk of complications, disability, and death in mothers and their infants. The objective of this study was to evaluate the current status of ANC emphasizing the number, timing, and content of examinations on a national scale.

**Methods:**

The data was collected from maternal and newborn’s health monitoring system at 8 provinces in China. After ethical approval, all pregnant women registered in the system at their first prenatal care visit, we included 49,084 pregnant women who had delivered between January 1, 2018 and December 31, 2018. Descriptive statistics of all study variables were calculated proportions and chi-square for categorical variables.

**Results:**

Of the 49,084 women included in this study, the mean number of ANC visits was 6.95 ± 3.45. By percentage, 78.79% women received ANC examinations at least five times, 39.93% of the women received ANC examinations at least eight times and 16.66% of the women received ANC examinations at least 11 times. The proportion of first ANC examination in first trimester was 61.87%. The percentage of normative ANC examinations and the percentage of qualified ANC examinations were 30.98 and 8.03% respectively. Only 49.40% of the total women received all six kinds of examination items in first ANC examination: 91.47% received a blood test, 91.62% received a urine test, 81.56% received a liver function examination, 80.52% received a renal function examination, 79.07% received a blood glucose test, and 86.66% received a HIV/HBV/syphilis tests. 50.85% women received the first ANC examination in maternal and child health care (MCH) institutions, 14.07% in a general hospital, 18.83% in a township hospital, 13.15% in a community health services center, and 3.08% in an unspecified place. The proportion of women who received each of the ANC examination items in community health services center was the highest, but that in the MCH institutions was the lowest.

**Conclusions:**

There is a big difference between the results of this study and the data in official reports, this study found the current status of antenatal care is not optimal in China, findings from this study suggest that the systematization, continuity and quality of ANC examinations need to be improved.

## Background

Within the continuum of reproductive health care, antenatal care (ANC) provides a platform for important health-care functions, including health promotion, screening and diagnosis, and disease prevention. It has been established that by implementing timely and appropriate evidence-based practices, ANC can save lives [1]. Reasonably frequent ANC visits at the appropriate gestational weeks can not only guarantee the safety of mother and child but also conform to the principles of health economics [2]. In addition, the proportion of women accessing ANC is the key indicator to measure the achievement of the fourth and fifth Millennium Development Goals, and improving that proportion involves tackling issues commonly associated with the Sustainable Development Goals [3, 4]. In 2016, WHO recommendations on antenatal care for a positive pregnancy experience recommended a woman see her health provider at least 8 times during her pregnancy to detect and manage potential problems and reduce the likelihood of a stillbirth or neonatal death [1]. WHO recommends that pregnant women have their first contact in the first 12 weeks’ gestation, with subsequent contacts taking place at 20, 26, 30, 34, 36, 38 and 40 weeks’ gestation [5]. WHO also suggested that ANC examination should include measuring blood pressure, as well as weight during pregnancy, urine test and blood test, etc. [1]. Besides clinical guidance, the new guidelines contain recommendations on health system interventions to improve the utilization and quality of antenatal care [5].

In order to improve the quality of ANC, the National Health Commission of the People’s Republic of China (NHC) also proposed guidelines and regulations in the last 20 years [6–9]. The Guidelines for Maternal Health Care Service (GMHCC) and the National Basic Public Health Service Project (BPHS) of China suggest that all pregnant women attend no less than five times at antenatal health services provided by formal medical institutions, including at least once during the first trimester, at least two times during the second trimester (16–20 weeks and 21–24 weeks), and two times during the third trimester (including at least one visit after 36 weeks of pregnancy). According to the GMHCC, the prescribed antenatal examinations should mainly include physical examination, obstetric examination, and laboratory assistant examinations such as blood test, urine test, liver function, renal function, hepatitis B test, syphilis test and HIV test [6, 7]. The guideline of pre-pregnancy and pregnancy care (2018) formulated by the Chinese Medical Association (CMA) recommends 7 ~ 11 ANC sessions, namely: 6–13 weeks ^+ 6^, 14–19 weeks ^+ 6^, 20–24 weeks, 25–28 weeks, 29–32 weeks, 33–36 weeks, 37–41 weeks. Pregnant women with high risk should increase the number of visits.

In China, as broadly outlined in the 1994 law of the People’s Republic of China on maternal and infant health care, the indicators for antenatal care utilization are: use of antenatal care (i.e., making at least one antenatal visit), initiating antenatal care during the first trimester of pregnancy (i.e., within the first 12 weeks of pregnancy), and making five or more antenatal care visits [10]. According to the Report on Women and Children’s Health Development in China (2019), the percentage of pregnant women who received ANC examinations was 96.6%, the registered percentage of women who received early pregnancy examinations was 92.5%, and the percentage of pregnant women who received more than five ANC examinations was 92.8%.

There is increasing global awareness that good quality care is key to keeping mothers and babies alive and well, the Sustainable Development Goals have set ambitious health-related targets for mothers, newborns, children under the umbrella of Universal Health Coverage by 2030. Addressing quality of care will be fundamental in reducing maternal and newborn mortality and achieving the health-related SDG targets [51]. Quality ANC includes access to ANC, the number of ANC visits, and the content of the ANC [11]. Some studies have showed that the utilization of ANC services was not optimal in terms of the frequency of attending, as well as the coverage of examination items [12, 13]. There may be problems of insufficient timeliness and low quality of ANC examinations behind the data of the apparent high examination rate, such as a late start of antenatal care visits [14, 15], or a lower than recommended number of visits [12, 16, 17], or incomplete examinations [17, 18]. The existing literature notes the following limitations: 1) There is no indicator system that reflects the continuity of antenatal care examinations; 2) The service quality behind the high rate of antenatal care examination needs to be further verified and analyzed; 3) There are few studies exploring the quality of antenatal care examinations. Therefore, it is urgent to evaluate the current status of ANC emphasizing the number, timing, and content of examinations on a national scale.

## Methods

### Data collection

The data of this study were drawn from Maternal and Newborn Health Monitoring System (MNHMS) set up by the National Center for Women and Children’s Health (NCWCH) for the Maternal and Newborn Health Monitoring Program (MNHMP) in 2013 [48]. The MNHMS was established to monitor the prenatal health care and pregnancy outcomes of pregnant women. In 2018, 16 districts/counties of eight provinces were covered in this survey. The eight provinces (with the selected districts) are: Hebei (Xinhua and Zhengding), Liaoning (Lishan and Tiedong), Hunan (Yueyanglou and Yueyang), Hubei (Macheng, Luotian), Fujian (Haicang and Jimei), Guangdong (Zijin and Longchuan), Sichuan (Gongjing and Rong County), and Yunnan (Tonghai and Huaning). Sixteen districts/counties of eight provinces are selected by using the convenience sampling method. All pregnant women who were residents or lived more than six months at these places were enrolled at their first antenatal care (ANC) visit [49], the information about their ANC during pregnancy was collected from the Maternal and Child Care Records in each district/county, and the information of delivery was collected from their delivery registrations. Prior to data collection, our study was approved by the Ethics Committee of National Center for Women and Children’s Health, Chinese Center for Disease Control and Prevention (No.FY2015–007). The study was exempt from informed consent because all the information in our study was collected from the Maternal and Child Care Record recorded by doctors in place of collecting data face-to-face with the subjects [49], and the data we used does not identify individuals. Finally, all the data is recorded in the MNHMS [48], providing an opportunity to explore the current status of antenatal care in a population-based sample. To ensure the quality of the information, the data collection system includes many logic checks to prevent wrong inputs. In addition, the staff of the NCWCH conduct field supervision on data accuracy every year. Subject supervisors are professionals from the local Women and Children’s Health (WCH) Institutions who are responsible for the training of data collection and quality control.

### Data cleaning

In the MNHMS, a total of 52,144 women (who delivered live births between January 1, 2018 and December 31, 2018) had received at least 1 prenatal examination during prenatal care. Women whose age, ethnicity, residence, education, was missing (3014 persons) and women whose gestational weeks and delivery information was missing (46 persons) were excluded. In the end, the data of 49,084 registered pregnant women were analyzed in this study.

### Measures

Based on geographical location and economic development level, Hebei, Fujian, Guangdong and Liaoning province represent the eastern region, Hunan and Hubei province represent the central region and Sichuan and Yunnan province represent the western region. In this study, demographic characteristics included districts (eastern, central, western), maternal age (< 25, 25–35, >35), type of residence (local, non-local), maternal education (junior high or lower, senior high, university or above, unknown). Past fertile history included gravidity (0, ≥1) and parity (0, ≥1). Pregnancy-labor history included normal pregnancy, abnormal pregnancy and missing. Abnormal pregnancy included abortion, fetal death, stillbirth, neonatal death and birth defects. Mode of delivery included vaginal delivery, cesarean. Based on WHO recommendations, the Guideline for Maternal Health Care Service (GMHCC) and National Basic Public Health Service Project (BPHS), indicators for assessing ANC coverage (antenatal visits five or more times, antenatal visits eight or more times, antenatal visits eleven or more times, antenatal visit at first/second/third trimester) and items of ANC (blood test, urine test, liver function, renal function, blood glucose and HIV/HBV/syphilis tests) were collected and included in the analysis, the denominator of the rate calculated in the article is the number of pregnant women surveyed.

In the setting performance evaluation indicators of BHPS, it is required that the proportion of pregnant women who had received more than five ANC examinations is more than 85%, but it only considered the number of antenatal examinations which cannot reflect the real situation and ensure the quality of ANC examination, and ignored the importance of the continuity and content of the examination. In accordance with WHO standard, pregnant women should receive the right care, at the right times. So, two indicators were used to measure the quality of antenatal care in our study: normative ANC and qualified ANC examination. *Normative ANC* means antenatal care of sufficient timing and number of visits according to guideline; *Qualified ANC* means that women with normative ANC receiving the appropriate content of care. In this study, *Normative ANC examination* is defined that pregnant women receive at least five ANC examinations in accord with the given time required in the BPHS rules. *Qualified ANC examination* is defined that pregnant women receive routine blood, routine urine, liver function, renal function, hepatitis B, syphilis and HIV tests at their first ANC examination, and receive routine blood and urine tests at the rest of the four ANC examinations. A woman is classified to the normative ANC examination if her care started in the first trimester and two ANC cares received during the second trimester (16–20 weeks and 21–24 weeks), and two cares received during the third trimester (including at least one visit after 36 weeks of pregnancy), the total number of visits she received was at least five. Relatively, a woman is classified to the qualified ANC if she received blood test, urine test, liver function, renal function, hepatitis B test, syphilis test and HIV test in the first trimester, and received blood test, urine test in the rest four given times based on normative ANC examination. The two indictors used to consider not only the right times pregnancy women should receive but also consider the right care pregnancy women should receive.

### Statistical analysis

Analysis of data was performed with the SPSS software (SPSS, version 18.0). A cutoff *P* value of < 0.05 was considered of statistical significance, and all *p*-values were bilateral. Descriptive statistics of all study variables were calculated proportions and chi-square for categorical variables.

## Result

Table 1 shows the characteristics of the 49,084 women included in this study. There was a significant difference between regions. 27,714 of the women (56.46%) came from the eastern region, 13,345 (27.19%) from the central region, and 8025 (16.35%) from the western region. The average age of these women was 28.76 ± 4.72 years old, the youngest woman was 12 years old and the oldest was 54 years old. By age cohort, 73.14% of these women were 25–35 years old, 9.14% of them were over 35 years old. Almost all (94.08%) of these women were local residents. With respect to education level, 29.47% of the participants had finished junior high or lower, 31.55% had received senior high school education, and 38.54% had college education or above. The education level of pregnant women varies in different regions: in the eastern region the majority (53.79%) of the women had college education or above, while in the central region the majority (55.88%) of women had completed senior high school education, and in the western region just under half (48.59%) of women had completed no more than junior high. Only 6030 (12.29%) women were in their first pregnancy, while 23,429 (47.73%) had given birth before, and the number of childbirths was up to eight. The percentage of women who had more than one childbirth was higher in the eastern region (55.83%) and in the western region (58.07%) than for those in the central region (24.70%). A total of 16,341 (33.29%) women had a history of abnormal pregnancy, and the proportion of women with a history of abnormal pregnancy was highest in the western region (50.28%). The proportion of caesarean deliveries was 45.66% of the total. The proportion of caesarean deliveries in the central region (53.11%) was significantly higher than in the eastern (44.81%) and western regions (36.21%).

**Table 1:** Basic information of pregnant women in different regions

Variables	Total	Eastern	Central	Western	χ^2^	*P*
	N(%,95CI)	N(%,95CI)	N(%,95CI)	N(%,95CI)		
Age (years old)					973.862	0.000
< 25	8700 (17.7, 17.4–18.1)	4064 (14.7,14.3–15.1)	2256 (16.9,16.3–17.5)	2380 (29.7,28.7–30.7)		
25–35	35,899 (73.1,72.8–73.5)	21,062 (76.0,75.5–76.5)	9790 (73.4,72.6–74.1)	5047 (62.9,61.8–64.0)		
>35	4485 (9.1,8.9–9.4)	2588 (9.3,9.0–9.7)	1299 (9.7,9.2–10.2)	598 (7.5,6.9–8.0)		
Type of residents					354.959	0.000
local	46,178 (94.1,93.9–94.3)	26,398 (95.3,95.0–95.5)	12,117 (90.8,90.3–91.3)	7663 (95.5,95.0–95.9)		
Non-local	2906 (5.9,5.7–6.1)	1316 (4.8,4.5–5.0)	1228 (9.2,8.7–9.7)	362 (4.5,4.1–5.0)		
Education					8892.494	0.000
Junior high or lower	14,463 (29.5,29.1–29.9)	7246 (26.2,25.6–26.7)	3534 (26.5,25.7–27.2)	3683 (45.9,44.8–47.0)		
Senior high	15,485 (31.6,31.1–32.0)	5546 (20.0,19.5–20.5)	7457 (55.9,55.0–56.7)	2482 (30.9,29.9–31.9)		
University or above	18,919 (38.5,38.1–39.0)	14,908 (53.8,53.2–54.4)	2158 (16.2,15.6–16.8)	1853 (23.1,22.2–24.0)		
Unknown	217 (0.44,0.38–0.50)	14 (0.05,0.02–0.08)	196 (1.5,1.3–1.7)	7 (0.09.0.02–0.15)		
Gravidity					300.133	0.000
0	6030 (12.3,12.0–12.6)	3628 (13.1,12.7–13.5)	1124 (8.4,7.9–8.9)	1278 (15.9,15.1–16.7)		
≥ 1	43,054 (87.7,87.4–88.0)	24,086 (86.9,86.5–87.3)	12,221 (91.6,91.1–92.1)	6747 (84.1,83.3–84.9)		
Parity					3910.217	0.000
0	25,655 (52.3,51.9–52.1)	12,241 (44.2,43.6–44.8)	10,049 (75.3,74.6–76.0)	3365 (41.9,40.9–43.0)		
≥ 1	23,429 (47.7,47.3–48.2)	15,473 (55.8,55.3–56.4)	3296 (24.7,24.0–25.4)	4660 (58.1,57.0–59.2)		
Pregnancy-labor history					5288.252	0.000
Normal pregnancy	11,301 (23.0,22.7–23.4)	6451 (23.3,22.8–23.8)	2367 (17.7,17.1–18.4)	2483 (30.9,29.9–32.0)		
Abnormal pregnancy	16,341 (33.3,32.9–33.7)	10,225 (36.9,36.3–37.5)	2081 (15.6,15.0–16.2)	4035 (50.3,49.2–51.4)		
Missing	21,442 (43.7,43.3–44.1)	11,038 (39.8,39.3–40.4)	8897 (66.7,65.9–67.5)	1507 (18.8,17.9–19.6)		
Mode of delivery					806.943	0.000
Vaginal delivery	26,596 (54.2,53.7–54.6)	15,287 (55.2,54.6–55.8)	6254 (46.9,46.0–47.7)	5055 (63.0,61.9–64.1)		
caesarean	22,414 (45.7,45.2–46.1)	12,420 (44.8,44.2–45.4)	7088 (53.1,52.3–54.0)	2906 (36.2,35.2–37.3)		
Missing	74 (0.15,0.12–0.19)	7 (0.03,0.01–0.04)	3 (0.02,0.002–0.05)	64 (0.80,0.60–0.99)		

Table 2 presents the results of the number, percentages and quality of the ANC visits received by pregnancy women in different regions. The mean number of ANC visits was 6.95 ± 3.45 with a range from 1 to 28 times. By percentage, 78.79% (38,675) women received ANC examinations at least five times. As for the WHO recommendation (eight or more ANC visits during the pregnancy), 39.93% (19,603) of the women received ANC examinations at least eight times and 16.66% (8178) of the women received ANC examinations at least 11 times. The median gestational weeks of first ANC examination was 12^+ 3^ weeks. The proportions of women who had ANC examinations rates in the first trimester(< 13 weeks), second trimester(< 28 weeks) and third trimester(≥28 weeks) were respectively 61.87%, 90.34%, 91.92%. The percentage of normative ANC examination and the percentage of qualified ANC examination were 30.98% and 8.03% of all examinations, respectively. After comparison, we can see the mean number, the percentage and the quality of the ANC received by women in the western region was significantly higher than in the central and eastern regions.

**Table 2:** Number and percentages of ANC visits received by pregnant women in different regions of China

Variables	Total	Eastern	Central	Western	χ^2^	*P*
	N(%,95CI)	N(%,95CI)	N(%,95CI)	N(%,95CI)		
Number of ANC visits						
≥ 5	38,675 (78.8,78.4–79.2)	20,208 (72.9,72.4–73.4)	10,615 (79.5,78.9–80.2)	7852 (97.8,97.5–98.2)	2320.45	0.000
≥ 8	19,603 (39.9,39.5–40.4)	10,101 (36.5,35.9–37.0)	2803 (21.0,20.3–21.7)	6699 (83.5,82.7–84.3)	8476.94	0.000
≥ 11	8178 (16.7,16.3–17.0)	3626 (13.0,12.7–13.5)	239 (1.8,1.6–2.0)	4313 (53.7,52.7–54.8)	10,328.50	0.000
Percentage of ANC visits, by trimester						
First trimester(< 13 weeks)	30,368 (61.9,61.4–62.3)	14,551 (52.5,51.9–53.1)	9104 (68.2,67.4–69.0)	6713 (83.7,82.8–84.5)	2872.43	0.000
Second trimester(< 28 weeks)	44,342 (90.3,90.1–90.6)	24,451 (88.2,87.9–88.6)	11,984 (89.8,89.3–90.3)	7907 (98.5,98.3–98.8)	763.02	0.000
Third trimester(≥28 weeks)	45,117 (91.9,91.7–92.2)	24,936 (90.0,89.6–90.3)	12,176 (91.2,90.8–91.7)	8005 (99.8,99.6–99.9)	811.68	0.000
Quality of ANC visits						
Normative ANC	15,207 (31.0,30.6–31.4)	5687 (20.5,20.0–21.0)	4601 (34.5,33.7–35.3)	4919 (61.3,60.2–62.4)	4943.51	0.000
Qualified ANC	3941 (8.0,7.8–8.3)	141 (0.5,0.4–0.6)	1212 (9.1,8.6–9.6)	2588 (32.3,31.2–33.3)	8517.59	0.000

Note: The first, second, and third trimester were defined as a gestational age less than 13 weeks, 13–27 weeks, and 28–42 weeks, respectively

Tables 3 and 4 show the distribution of items in first ANC examination by different regions and different type of institutions. Only 49.40% of the total women received all six kinds of examination items in first ANC examination: 91.47% received a blood test, 91.62% received a urine test, 81.56% received a liver function examination, 80.52% received a renal function examination, 79.07% received a blood glucose test, and 86.66% received HIV/HBV/syphilis tests. The proportion of women who were checked for all items at the first ANC examination in the western region was significantly higher than in the central and eastern regions. By place of examination, 24,960 (50.85%) women received the first ANC examination in  a maternal and child health care (MCH) institution, 6910 (14.07%) in a general hospital, 9245 (18.83%) in a township hospital, 6459 (13.15%) in a community health services center, and 1510 (3.08%) in an unspecified place. The proportion of women who received each of the ANC examination items in community health services center was the highest, but that in the MCH institutions was the lowest.

**Table 3:** Distribution of items tested in first ANC examination by regions

Variables	Total	Eastern	Central	Western	χ^2^	*P*
	N(%,95CI)	N(%,95CI)	N(%,95CI)	N(%,95CI)		
Blood test	44,896 (91.5,91.2–91.7)	24,938 (90.0,89.6–90.3)	12,060 (90.4,89.9–90.9)	7898 (98.4,98.1–98.7)	984.392	0.000
Urine test	44,972 (91.6,91.4–91.9)	23,936 (86.4,86.0–86.8)	13,222 (99.1,98.9–99.2)	7814 (97.4,97.0–97.7)	2308.864	0.000
Liver function	40,035 (81.6,81.2–81.9)	21,364 (77.1,76.6–77.6)	10,860 (81.4,80.7–82.0)	7811 (97.3,97.0–97.7)	9724.011	0.000
Renal function	39,520 (80.5,80.2–80.9)	20,963 (75.6,75.1–76.2)	10,759 (80.6,80.0–81.3)	7798 (97.2,96.8–97.5)	2143.910	0.000
Blood glucose	38,813 (79.1,78.7–79.4)	21,446 (77.4,76.9–77.9)	9602 (72.0,71.2–72.7)	7765 (96.8,96.4–97.2)	2647.125	0.000
HIV/HBV/syphilis tests	42,537 (86.7,86.4–87.0)	23,453 (84.6,84.2–85.1)	11,134 (83.4,82.8–84.1)	7950 (99.1,98.9–99.3)	1496.736	0.000
All six items	24,248 (49.4,49.0–49.8)	11,058 (39.9,39.3–40.5)	6865 (51.4,50.6–52.3)	6325 (78.8,77.9–79.7)	3800.857	0.000

**Table 4:** Distribution of items tested at the first ANC examination by type of institutions

Variables	MCH	General hospital	Township hospital	Community health services center	unknown	χ^2^	*P*
	N(%,95CI)	N(%,95CI)	N(%,95CI)	N(%,95CI)	N(%,95CI)		
Blood test	22,266 (89.2,88.8–89.6)	6208 (89.8,89.1–90.6)	8480 (91.7,91.2–92.3)	6447 (99.8,99.7–99.9)	1495 (99.0,98.5–99.5)	930.099	0.000
Urine test	21,951 (87.9,87.5–88.4)	6432 (93.1,92.5–93.7)	8742 (94.6,94.1–95.0)	6385 (98.9,98.6–99.1)	1462 (96.8,95.9–97.7)	1056.174	0.000
Liver function	18,731 (75.0,74.5–75.6)	5602 (81.1,80.2–82.0)	7914 (85.6,84.9–86.3)	6401 (99.1,98.9–99.3)	1387 (91.9,90.5–93.2)	3754.487	0.000
Renal function	18,418 (73.8,73.2–74.3)	5566 (80.6,79.6–81.5)	7776 (84.1,83.4–84.9)	6386 (98.9,98.6–99.1)	1374 (91.0,89.6–92.4)	2780.063	0.000
Blood glucose	18,574 (74.4,73.9–75.0)	5460 (79.0,78.1–80.0)	7258 (78.5,77.7–79.3)	6096 (94.4,93.8–94.9)	1425 (94.4,93.2–95.5)	1579.448	0.000
HIV/HBV/syphilis tests	20,921 (83.8,83.4–84.3)	5680 (82.2,81.3–83.1)	8026 (86.8,86.1–87.5)	6416 (99.3,99.1–99.5)	1494 (98.9,98.4–99.5)	1543.362	0.000
All six items	9433 (37.8,37.2–38.4)	3657 (52.9,51.8–54.1)	5296 (57.3,56.3–58.3)	4745 (73.5,72.4–74.5)	1117 (74.0,71.8–76.2)	3470.674	0.000

Table 5 shows the distribution of blood test and urine test administered in different pregnancy periods and different regions. Among the 30,368 women in first trimester, 93.64% received a blood test. The proportion was highest in the western region (98.64%). A urine test was given to 92.51% of the women in the first trimester. The proportion in the central region was the highest (98.82%). Among the 44,342 women in second trimester, 77.20% received a blood test. The proportion in the western region was the highest (87.64%). 55.97% of women in the second trimester received a urine test and the proportion in the western region was the highest (79.07%). Among the 45,117 women in third trimester, 73.66% received a blood test and the proportion in the western region was the highest (90.88%). Of the women in the third trimester, 34.54% received a urine test and the proportion in the western region was the highest (83.89%).

**Table 5:** Distribution of Blood test and Urine test by trimester and region

Variables	Blood test				χ^2^	*P*	Urine test				χ^2^	*P*
	Eastern	Central	Western	Total			Eastern	Central	Western	Total		
	N(%,95CI)	N(%,95CI)	N(%,95CI)	N(%,95CI)			N(%,95CI)	N(%,95CI)	N(%,95CI)	N(%,95CI)		
First trimester	13,247 (91.0,90.6–91.5)	8568 (94.1,93.6–94.6)	6622 (98.6,98.4–98.9)	28,437 (93.6,93.4–93.9)	607.054	0.000	12,534 (86.1,85.6–86.7)	8997 (98.8,98.6–99.1)	6563 (97.8,97.4–98.1)	28,094 (92.5,92.2–92.8)	1644.438	0.000
Second trimester	21,631 (78.1,77.6–78.5)	9227 (69.1,68.4–69.9)	7033 (87.6,86.9–88.4)	37,891 (77.2,76.8–77.6)	1000.368	0.000	12,551 (45.3,44.7–45.9)	8574 (64.3,63.4–65.1)	6345 (79.1,78.2–80.0)	27,470 (56.0,55.5–56.4)	3391.361	0.000
Third trimester	19,926 (71.9,71.4–72.4)	8936 (67.0,66.2–67.8)	7293 (90.9,90.3–91.5)	36,155 (73.7,73.3–74.1)	1579.198	0.000	4209 (15.2,14.8–15.6)	6011 (45.0,44.2–45.9)	6732 (83.9,83.1–84.7)	16,952 (34.5,34.1–35.0)	13,885.87	0.000

## Discussion

### There is a big difference between the results of this study and the data in official reports

According to the Report on Women and Children’s Health Development in China (2019), the ANC coverage increased from 83.7% in 1996 to 96.6% in 2018 [19], the registered proportion of women receiving early pregnancy examination was 92.5%, and the proportion of pregnant women who had received more than five ANC examinations was 92.8% in 2018. However, this study suggests that the proportion of women who received early pregnancy examination was only 61.87%, and only 78.79% of the women received at least five examinations. The proportion of women who received a normalized ANC examination or a qualified ANC examination was only 30.98 and 8.03% respectively. These indicators were significantly lower than the official reported data. The reasons for these discrepancies may be the difference of statistical standards and the data sources. First, the data in the National Annual Report on Maternal and Child health was based on the national annual system for Maternal and Child Health which was established in the 1990s. The indicators collected by the system were based on statistical data collected at all levels, rather than the individual cases. Secondly, the statistics considered only the number of antenatal examination and the trimester when the first one was carried out, and ignored the importance of the time of subsequent examinations and the content of the examinations. There were no related data or indicators to determine whether the ANC examinations were carried out in accordance with the distribution of examinations over the trimesters and the examination contents required by the GMHCC, which gives an incomplete picture of the situation. Our study monitored the prenatal health care and pregnancy outcomes of pregnant women from their first antenatal care visit, and the items addressed in each ANC examination (including physical examination and laboratory assistant examinations) during pregnancy. The information was collected in each district/county continuously and systematically, to obtain more accurate and reliable data reflecting the health status and the management of pregnant women in China as a form of strict quality control. At the same time, two indicators were used to comprehensively reflect the actual number, timeliness and standardization of the ANC examination contents in the monitoring area: normative ANC and qualified ANC examination. In this paper, the definition of normative  and qualified  ANC  examinations is more rigorous. In addition to the total number of ANC examinations during pregnancy at least 5 times, normative  ANC examination also require pregnant women to receive prenatal examinations in accordance with the national basic public health service standards within each specified time. Qualified ANC examination requires that on the basis of normative examination, blood pressure hemoglobin test and other tests are carried out in each  ANC examination.

### The current status of antenatal care is not optimal

The number, timing and the content of ANC examination services are particularly important in identifying the risks of pregnancy and management of labor complications [20]. China has made impressive progress in maternal and child survival in the past 20 years [21]. During the past decades, the antenatal examination rate increased rapidly [22], although the percentage of women who have had at least one ANC examination has stayed pretty high [23], the percentage of women who receive prescribed ANC examinations (normative ANC examination and qualified ANC examination) which conform to the GMHCC of China is still low. Thus the current status of antenatal care is not optimal. Our results are supported by another two studies: An epidemiological study [16] on compliance with antenatal care in Tongzhou District of Beijing in 2017 found that the proportion of women who regularly received ANC (45.2%)was much lower than the proportion of women who received at least five ANC examinations (79.3%). The simple calculation of the number of ANC examinations had little significance for the evaluation of the status of ANC. Chen’s [18] study on coverage, regional disparity and equity of key maternal and child health interventions in China also had similiar results. He found that the proportion of women who received at least one ANC examination was 95%, but the proportions of those who received at least four or five ANC examinations, and of those whose examinations met the basic quality standards of ANC were only 66% (4 examinations), 52% (5 examinations) and 62% (basic standards), while the proportion of women whose examinations met both of these conditions (received at least 5 examinations that met the basic quality standards of ANC) was even lower, only 39.08%. Although the proportion of those with at least one ANC examination was high, the quality of antenatal care was suboptimal with wide regional disparity.

### The systematization and continuity of ANC examinations need to be improved

Antenatal care for first time mothers is key, since it will determine how they use antenatal care in future pregnancies [5]. The timing of ANC during the first trimester highlights the importance of early pregnancy tests. It is recommended that women should initiate ANC examinations as early as possible, at no later than 12 weeks of gestation [24]. Initiating antenatal care during the first trimester can also reduce poor pregnancy outcomes [25, 26]. In our study the median gestational weeks of first ANC examination was 12^+ 3^ weeks, but the proportion of first ANC examination in first trimester in this study was only 61.87%, which means some vulnerable women’s needs in early antenatal care aren’t being met. On the basis of whole-process health service, although early, middle and late use can be classified according to the timing of the ANC examination, women who access antenatal care early in their pregnancy are able to receive timely information concerning the full range of antenatal screening tests available [27], while women who initiate antenatal care after the first trimester may lose the opportunity to benefit from these same screening tests [28]. But having ANC examination in the first trimester also does not guarantee a woman’s continued use of a full range of standard ANC throughout the pregnancy [18]. In 2011, The NHC that proposed a quantitative standard [29] of at least five ANC visits, required at least two visits in each of the first, second and third trimesters. To a certain extent, this is an indicator of continuity of health care services during pregnancy. In this study, the percentage of normative ANC examination calculated according to the time specification was only 30.98% of the total, which means the systematization and continuity of ANC examinations needs to be improved.

### The quality of ANC examination needs to be improved

More and better quality contacts between all women and their health providers throughout pregnancy facilitates the uptake of preventive measures, timely detection of risks, reduces complications and addresses health inequalities [5]. The expected benefits of antenatal care visits depend very much on the contents of the antenatal examinations [30]. This study examined the association of items in the examinations with the ANC checklist items recommended by the GMHCC (blood test, urine test, liver function, renal function, blood glucose and HIV/HBV/syphilis tests). The results showed that the proportion of women who were examined for each of the six basic items in first trimester was more than 75%, but only 49.4% of the women received all 6 kinds of examination items. This reminds us that even if pregnant women have received prenatal care, some pregnant women still do not receive the basic quality of ANC service, which suggests that the service providers pay more attention to the quantity and ignore the quality and content of the services. According to the requirements of GMHCC, routine blood and urine tests must be performed at the prescribed five antenatal examinations, and auxiliary examination items should be appropriately added according to need. In this study, the percentages of blood tests and urine tests were calculated by trimester. The results showed that as the gestational age increases, the percentage of women who receive blood and urine tests gradually decreases, and the percentages for blood tests (73.66%) and urine tests (34.54%) are lowest in the third trimester. The percentage of qualified ANC examination, which  is defined according to time and test items, was only 8.03% of the total, indicating that most pregnant women in the monitoring area did not receive a qualified ANC examination, and the quality of ANC examinations needs to be improved urgently. From the supply-side perspective, the percentage of women who received all 6 kinds of examination items in the first antenatal care visit was highest in community health centers while lowest in MCH. An analysis of possible reasons suggests several points. Firstly, we think the key of success behind community health services center perform ANC examinations very well compared to all other providers is the government financial support. China comprehensively implemented the national project to provide BPHS since 2009, special central financial transfer payment to carry out essential public health services all over is a strong support, the national per capita funding for BHPS increased from 15yuan in 2009 to 74yuan in 2020, funds for BPHS projects have become an important source of income for primary medical institutions. The government afforded all the costs of antenatal examinations and antenatal visits. The community health center, township health center and mothers themselves did not have to bear any cost [50]. In addition, BPHS funds are required to be allocated according to the quantity and quality of services, this approach not only reduced the financial pressures on primary medical institutions but also made providers and medical staffs more motivated to provide high quality maternal services. However, pregnant women need to pay for antenatal examinations in other medical institutions, such as general hospitals and MCH, because these institutions do not enjoy BPHS funds. Therefore, some examinations are optional for pregnant women. To further improve the quality of ANC service, other provider and health policy researchers should suggest government to change the investment strategy and convert the previous investment from providers to investment on demand, to make sure pregnant women can freely choose any medical institution within their jurisdiction to accept free antenatal care services without paying any costs. Second, the inconvenience of long distance travel to a medical institution might affect women’s ANC services seeking behaviors [31]. Gage’s [32] study showed that poor road conditions significantly reduced the likelihood of timely receipt of antenatal care and of four or more antenatal care visits, while the availability of a health center within five kilometers significantly increases the odds of each outcome. This finding suggests that improving the use of ANC services would require improvement of the availability of services. Community health centers are to serve the communities, families and residents [47]. This characteristic determines that the accessability of its services is higher than that of other medical institutions.

### The capacity of antenatal care service in western China has been significantly improved

Previous studies have reported that in economically developed eastern China, both quantity and quality of ANC were somewhat better than in other regions [17, 33, 34]. But our study has different findings: the percentage of women in the *western* region who had at least five ANC examinations, normative ANC examination or qualified ANC examination was higher than in other regions. The possible reasons for this finding could be evidence that remarkable results the country has achieved in improving maternal and child health in western China. Maternal health has been set as a priority public health issue in the western region and ANC examinations are provided in the western area as part of the national strategy to reduce the number of maternal deaths. Since the launch of the deepening reform of the healthcare system in 2009 [35], the central government has increased financial support for the implementation of national basic public health services projects in the western region [36]. In 2011, the central government’s financial investment of 43 billion yuan to implement the capacity building project in mid-west county MCH institutions has equipped these institutions with basic equipment. In 2013, the NHFPC launched the MCH capacity improvement project in the western region, which strengthened and improved the MCH work in the western region from three aspects: enhancing personnel capacity, improving hardware conditions and establishing a long-term cooperation mechanism [37]. Some previous studies have also showed that ANC services have improved in the western region in recent years. Zhang’s [38] study found that the level of utilization of ANC services among urban and rural pregnant women in Gansu has generally improved, since 93.0% of pregnant women took the first examination within the first 12 weeks. This is higher than Liu’s [39] 2011 study in 45 western counties, where 66.9% of women had an examination in the first trimester. Zhu’s [40] study in 2015 showed that 30.2% of pregnant women in rural areas of Shanxi province completed all the required examinations, which was above the national average of 29.1%. In addition, previous studies have explored determinants of ANC use among pregnant women [41–46]. An analysis of the demographic characteristics of the pregnant women in this survey reveals that most pregnant women in the western region were local residents with a low education level. The proportion of pregnant women with a history of gravidity and parity was high, and the proportion of abnormal pregnancy-labor outcomes in the western region is far higher than in other regions. Thus women in the western region may pay more attention to antenatal examinations, so that the compliance with ANC recommendations is better than in other regions. This might be because women with these characteristics worry whether they might suffer complication during pregnancy or delivery. This leads these women to participate in more examinations than others. What’s more, given this kind of pregnant women, doctors would encourage them to take ANC examinations more carefully and frequently.

Our study has several strengths worth considering. First, using a relatively large sample is one of the major strengths of this study. Second, as above mentioned, we use two indicators-- normative ANC and qualified ANC examination, which are good predictors for giving a comprehensive description of the situation of ANC in China. Third, the data we used is monitoring data so that there was no recall bias. Fourth, our study analyses the ANC utilization in monitored regions of China from the perspective of demand-side and supply-side. Nevertheless, despite the strengths, there are inevitably some limitations that should be considered: First, because this study is based on the analysis of the case data reported in MNHMS, there may be some pregnant women whose ANC examinations have not been reported so that the assessment of ANC in the monitored regions is incomplete. And women with other birth outcomes (miscarriages) and with zero ANC visits are not in the sample which is also important for the evaluation of ANC examinations because ANC is linked to birth outcomes to some extent. The association between the quality of antenatal care examinations and birth outcomes needs to be investigated in our further work. There is certain selection bias in the selection of research objects based on the current database, we will make further study to help make up. Second, some high-risk indicators and monitoring data of antenatal examination items in our study, such as blood test, urine test, liver function, renal function, blood glucose and HIV/HBV/syphilis tests, were not complete. After reporting the data of all examination items, the women with missing items are listed by default as not having done this examination, so the percentage of women who received an examination of some items may be low. Third, other factors or contents we did not consider may have important influence on the frequency and quality of antenatal examinations, especially the screening and evaluation of high-risk factors during pregnancy. Our study will continue to follow up and get future analyses. Fourth, in view of the current data, our study did not include women’s perspective of ANC quality in the measure of quality of care, this is a major limitation needs to be included as a consideration in our further work.

## Conclusions

This study shows that the proportion of women who received early pregnancy examination was only 61.87%, and only 78.79% of the women received at least five examinations, two indicators which comprehensively reflected the actual number, timeliness continuity and standardization of the ANC examination contents were used to measure the quality of antenatal care: normative ANC and qualified ANC examination were only 30.98 and 8.03% respectively. These indicators were significantly lower than the official reported data, it is clearly showed that the current status of antenatal care is not optimal in China; the systematization, continuity and quality of ANC examinations need to be improved. And, of course, we can see that the remarkable results the country has achieved in improving maternal and child health in western China: the capacity of ANC service in western China has been significantly improved.

## Data Availability

All data generated or analysed during this study are included in this published article.
